# Development of a brain-computer interface for patients in the critical care setting

**DOI:** 10.1371/journal.pone.0245540

**Published:** 2021-01-22

**Authors:** Andrey Eliseyev, Ian Jerome Gonzales, Anh Le, Kevin Doyle, Jennifer Egbebike, Angela Velazquez, Sachin Agarwal, David Roh, Soojin Park, E. Sander Connolly, Jan Claassen

**Affiliations:** 1 Department of Neurology, Columbia University Medical Center, New York, NY, United States of America; 2 Department of Neurosurgery, Columbia University Medical Center, New York, NY, United States of America; University of California Irvine, UNITED STATES

## Abstract

**Objective:**

Behaviorally unresponsive patients in intensive care units (ICU) are unable to consistently and effectively communicate their most fundamental physical needs. Brain-Computer Interface (BCI) technology has been established in the clinical context, but faces challenges in the critical care environment. Contrary to cue-based BCIs, which allow activation only during pre-determined periods of time, self-paced BCI systems empower patients to interact with others at any time. The study aims to develop a self-paced BCI for patients in the intensive care unit.

**Methods:**

BCI experiments were conducted in 18 ICU patients and 5 healthy volunteers. The proposed self-paced BCI system analyzes EEG activity from patients while these are asked to control a beeping tone by performing a motor task (i.e., opening and closing a hand). Signal decoding is performed in real time and auditory feedback given via headphones. Performance of the BCI system was judged based on correlation between the optimal and the observed performance.

**Results:**

All 5 healthy volunteers were able to successfully perform the BCI task, compared to chance alone (*p*<0.001). 5 of 14 (36%) conscious ICU patients were able to perform the BCI task. One of these 5 patients was quadriplegic and controlled the BCI system without any hand movements. None of the 4 unconscious patients were able to perform the BCI task.

**Conclusions:**

More than one third of conscious ICU patients and all healthy volunteers were able to gain control over the self-paced BCI system. The initial 4 unconscious patients were not. Future studies will focus on studying the ability of behaviorally unresponsive patients with cognitive motor dissociation to control the self-paced BCI system.

## Introduction

Disorders of consciousness are common with or without acute brain injury. Recent studies of acute brain injuries have identified behaviorally unresponsive patients that show evidence of volitional brain activation detected by functional magnetic resonance imaging (fMRI) or electroencephalography (EEG) [1, 2]. Patients that demonstrate this activation are in a state called cognitive motor dissociation (CMD), which has been associated in one study with later behavioral recovery of consciousness and functional outcomes [1]. These patients currently have no ability to communicate with loved ones or health care providers despite possessing some level of preserved consciousness. A common challenge for patients is their inability to consistently and effectively communicate their most fundamental physical needs [3], such as unrecognized pain, discomfort, feelings of loss of control and insecurity, depersonalization, anxiety, sleep disturbances, fear, and frustration [4]. Non-vocal techniques such as lip reading and gestures are the primary means of communication for these patients. These methods are, however, often inadequate for effective communication [5].

Brain-computer interface (BCI) technology translates cerebral electrical activity, typically recorded by EEG, into computer commands bypassing other body functions [6, 7]. Despite having used BCI systems efficiently for rehabilitation purposes [8, 9], their introduction in the critical care context is limited [10–12]. Implementation of BCI systems in critical care settings has faced a number of technical and logistical challenges. These challenges include low reliability due to auditory or physical distractions, possible extinction of goal-directed thinking, and fatigue [13]. Other challenges include physical disabilities. Patients, for example, might be unable to successfully use visual-based BCI systems due to eyelid apraxia or other visual impairments. Utilizing systems based on tactile input may also pose challenges, due to pain medication, extended bedrest, and skin breakdown. Auditory-based BCI tasks may be most promising, but are not without their own set of challenges. Auditory BCI studies with critically ill patients have often reported high variability and/or poor performance in a single session [12]. Multiple sessions pose challenges in a hectic ICU setting. Given this, currently studied BCI systems in the intensive care unit (ICU) focus on quick and reliable signaling (e.g., ‘yes’/‘no’ binary signals [11], steady-state visual evoked potential- (SSVEP-) based communication [10], or transient evoked potentials (P300 or N200) [12]) rather than spelling of words or sentences.

All these systems are cue-based (synchronous) BCI systems that are limiting to patients, as they are active only during pre-determined periods of when the patient is presented a cue. Users will only be able to generate a response during these pre-specific periods. Self-paced (asynchronous) systems are always active and analyze brain activity from continuously recorded data. These systems allow patients to freely activate the BCI at their own intention. This approach is particularly promising, as acutely brain injured patients often have a fluctuating mental status and might be unable to perform using a cue-based approach at the one pre-specified moment in time. Self-paced BCI systems, however, possess their own set of challenges. Due to the large amount of data continuously processed over time, self-paced BCI systems must rely on high true positive rates and a low number of false positive detections [14].

The overall goal of this study was to develop and test a prototype of a self-paced BCI system that can be employed in the critical care context that would provide the foundation to study behaviorally unresponsive patients in the future. Unresponsiveness was defined purely on behavior for the purposes of this study in other words, all patients that did not show any evidence of command following with a motor response (e.g., showing two fingers when asked to do so) were classified as unresponsive [15]. Patients were carefully assessed for the presence of locked in syndrome but this classification has obvious limitations in patients in complete locked in syndrome or those in cognitive motor dissociation [1]. However, this classification reflects the practice of current clinical practice outside of the research context.

## Methods

### Patient and healthy volunteer population

The study was approved by the Columbia University institutional review board. Approval number AAAS3851. Written consent was obtained from the patient or the designated surrogate for patients that were unable to provide consent themselves, and from healthy volunteers. We included adult patients who were admitted to the neurological intensive care unit between September 2019 and February 2020. Patients qualified for inclusion in the study if they had EEG placed to rule out electrographic seizures and a board certified epileptologist had determined that seizures were not seen. Additionally, patients qualified that were admitted to the neurocritical intensive care unit that did not have EEG placed but consented to have EEG recorded for research purposes alone. These patients only qualified for inclusion in the study if no seizures were recorded after review of the EEG placed for research purposes. Classification into conscious versus unconscious patients was based on the Command Following Score (CFS) [1, 15], which determines the ability of the patient to follow a verbal command with a motor response (e.g., showing two fingers or sticking out the tongue). We assessed for mimicking to minimize the risk of aphasia confounding the assessment of consciousness as explained in detail previously [15]. All patients with ongoing seizure activity were excluded. Healthy volunteers had EEG placed for research purposes alone.

### Experimental design

**Fig 1:**
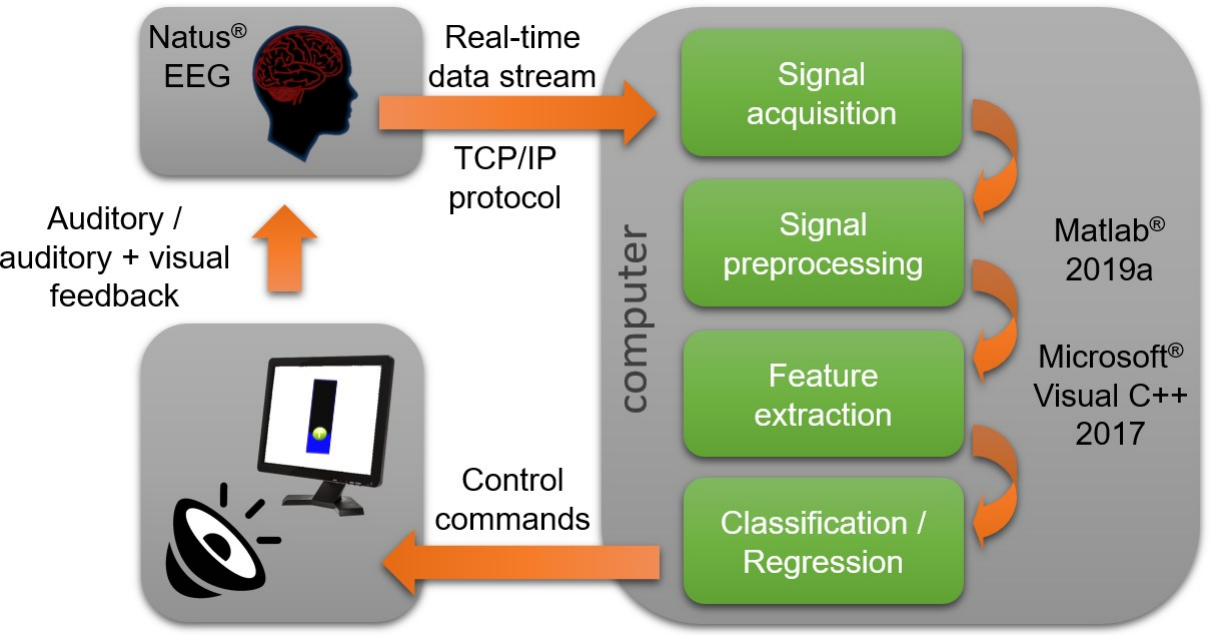
General scheme of the BCI system used in the experiment.

The BCI system utilized EEG signal acquisition from the subject with signal decoding occurring in a bedside computer, and sensory feedback provided to the patient (Fig 1). For all participants, 21 EEG electrodes (C3, C4, O1, O2, A1, A2, Cz, F3, F4, F7, F8, Fz, Fp1, Fp2, P3, P4, Pz, T7, T8, P7, P8) were applied with collodion to the patient's head by certified EEG technicians according to the International 10–20 system. EEG was obtained using unipolar channels via a standard clinical digital bedside video EEG monitoring system (XLTEK, Excel-Tech Corp., Natus Medical Incorporated, Oakville, Ontario, Canada; low-pass filter = 70 Hz, high-pass = 1 Hz, sampling rate = 200 Hz). Signal quality was visually checked during the experiment. EEG was transferred to the bedside decoding computer (BCI computer) via a TCP/IP protocol, developed in Microsoft^®^ Visual C++ 2017 (The Microsoft Corporate, Redmond, US). The EEG data decoder was implemented in Matlab^®^ 2019a (The MathWorks, Inc., Natick, US) and Microsoft^®^ Visual C++ 2017 providing real-time processing on a standard computer (Intel^®^ Xeon^®^ CPUE3-1245v5, 3.5 GHz, RAM 64 Gb) installed on a movable cart.

Auditory feedback was delivered using disposable earphones (E-A-RTONE™ 3A or 5A Insert Earphones) connected to the BCI computer. To provide visual feedback, a separate monitor was placed in front of the patient and connected to the BCI computer. A second monitor was connected to the BCI computer. This monitor was not visible to the patient and was used by the operator to control the experiment. The BCI computer allows the operator to tune a set of decoding algorithm hyper-parameters in real-time to optimize BCI performance [16]. The commands to the patients were given in English or Spanish, depending on the patient’s native language.

### Experimental protocol

**Fig 2:**
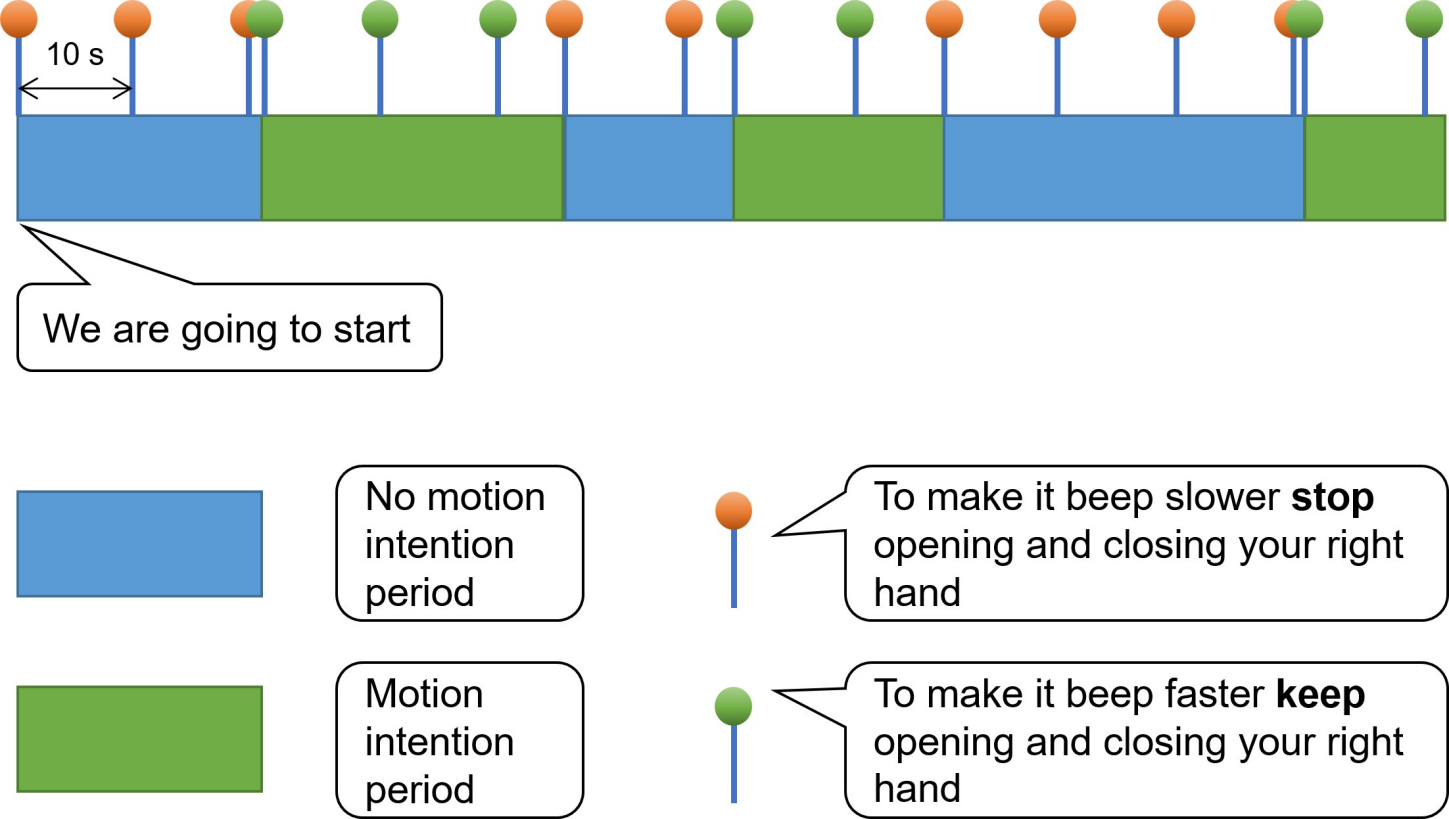
BCI protocol. To begin the session, each patient was first presented with the command ‘We are going to start.’ After the command ‘To make it beep faster keep opening and closing your right hand’, the patient was expected to try moving his/her right hand. After the command ‘To make it beep slower stop opening and closing your right hand’, the patient was expected to stop his/her right-hand motion. To ensure patients did not forget the task, each command was repeated every 10 seconds. Duration of each motion/no motion period was randomly chosen from the interval [15, 50] seconds.

**Fig 3:**
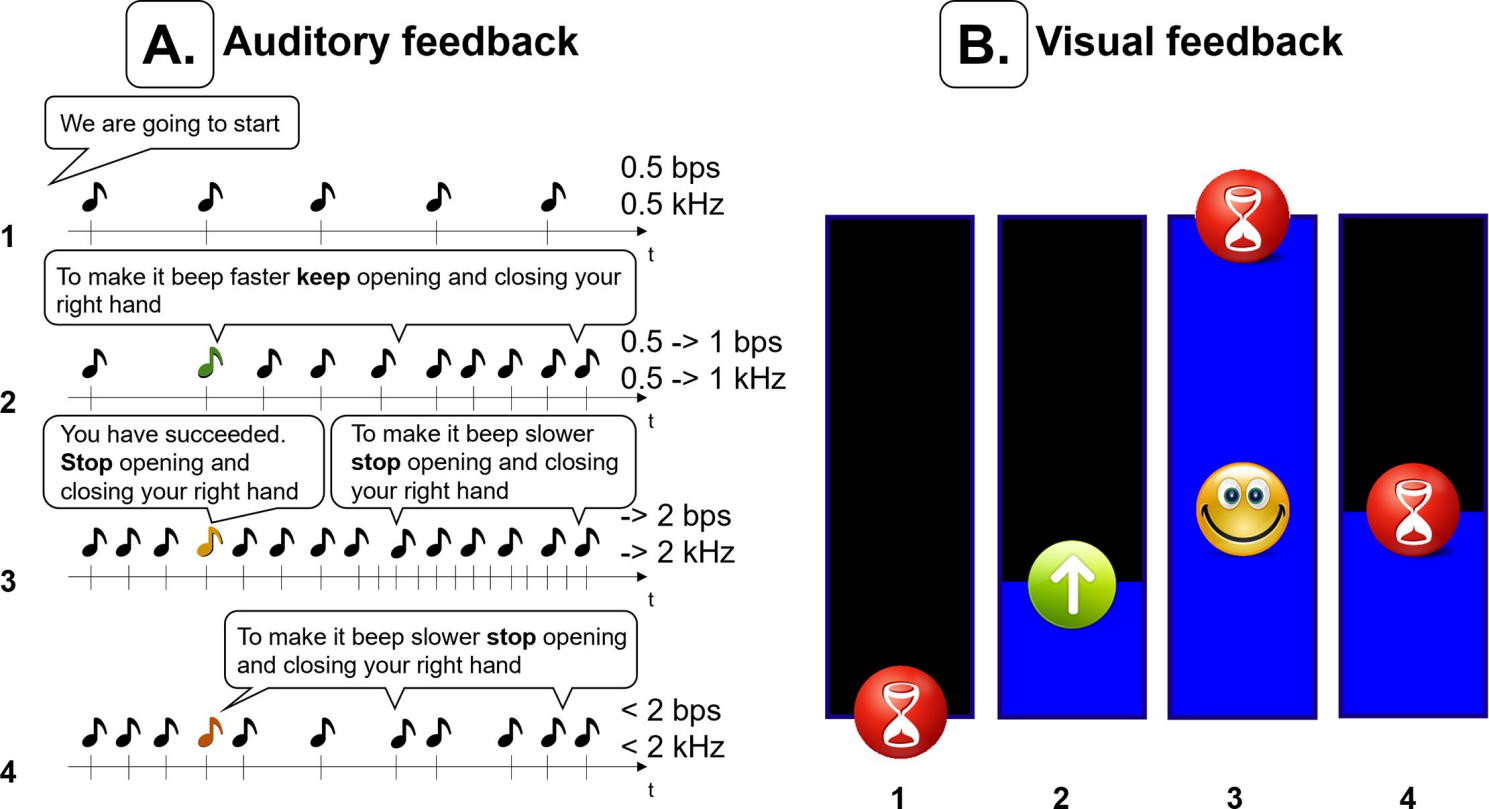
Auditory and visual feedback. (A) Auditory feedback. Each time the motion intention pattern was detected in the EEG signal, the pace of the sound (beets per second, bps) and sound frequency were increased (Δbps = 0.15, ΔkHz = 0.15) until 2 bps and 2 kHz were reached. If the pattern was not detected, both were decreased (Δbps = -0.15, ΔkHz = -0.15) until reaching 0.5 bps and 0.5 kHz. When reaching a pace of 2 bps and a 2 kHz frequency during the motion task the auditory feedback “you have succeeded” was provided to the patient. (B) Visual feedback. Each time the motion intention was detected, the size of the bar was increased by 0.1 until reaching of 1. Otherwise, it was decreased by 0.1, until reaching 0. Motion intention periods were indicated with a green arrow pointing up and a red hourglass was pictured when no motion intention patterns were detected. A smiley face was displayed when the bar reached 1 (full size).

Depending on the level of patient fatigue, the first or the first and the second session of the experiment were used for BCI model calibration (BCI training stage) and lasted 5 minutes per session. The last session was used for validation of the BCI model (BCI testing stage) and lasted 10 minutes. 5-minute breaks were provided between sessions. Patients were asked to control both the tempo of a beeping sound (Fig 2) and move a bar on a screen in order to provide both, an auditory and a visual task (Fig 3). As many ICU patients have visual apraxia and behaviorally unresponsive patients often have their eyes closed, we removed the visual feedback after completing the first 3 patients and the first 2 healthy volunteers. Following the auditory prompts, patients were asked to repeatedly open and close their right hand until hearing the “stop” command. The BCI system analyzed the corresponding EEG signal to detect hand motion every 100 ms. Each EEG detection or failure of detection of hand movement was reflected in the auditory or visual feedback provided to the patient. To prevent patient frustration caused by false detections, algorithm predictions were partially corrected with ground truth information during the calibration session. Ground truth in this context refers to the command to move or not to move that the patient was asked to perform at the moment. The weight of ground truth linearly decreased over time to 0 within 3 minutes to allow the patient to gain full control over the BCI system. During the validation session, no ground truth information was provided to the algorithm.

### Feature extraction and data formation

**Fig 4:**
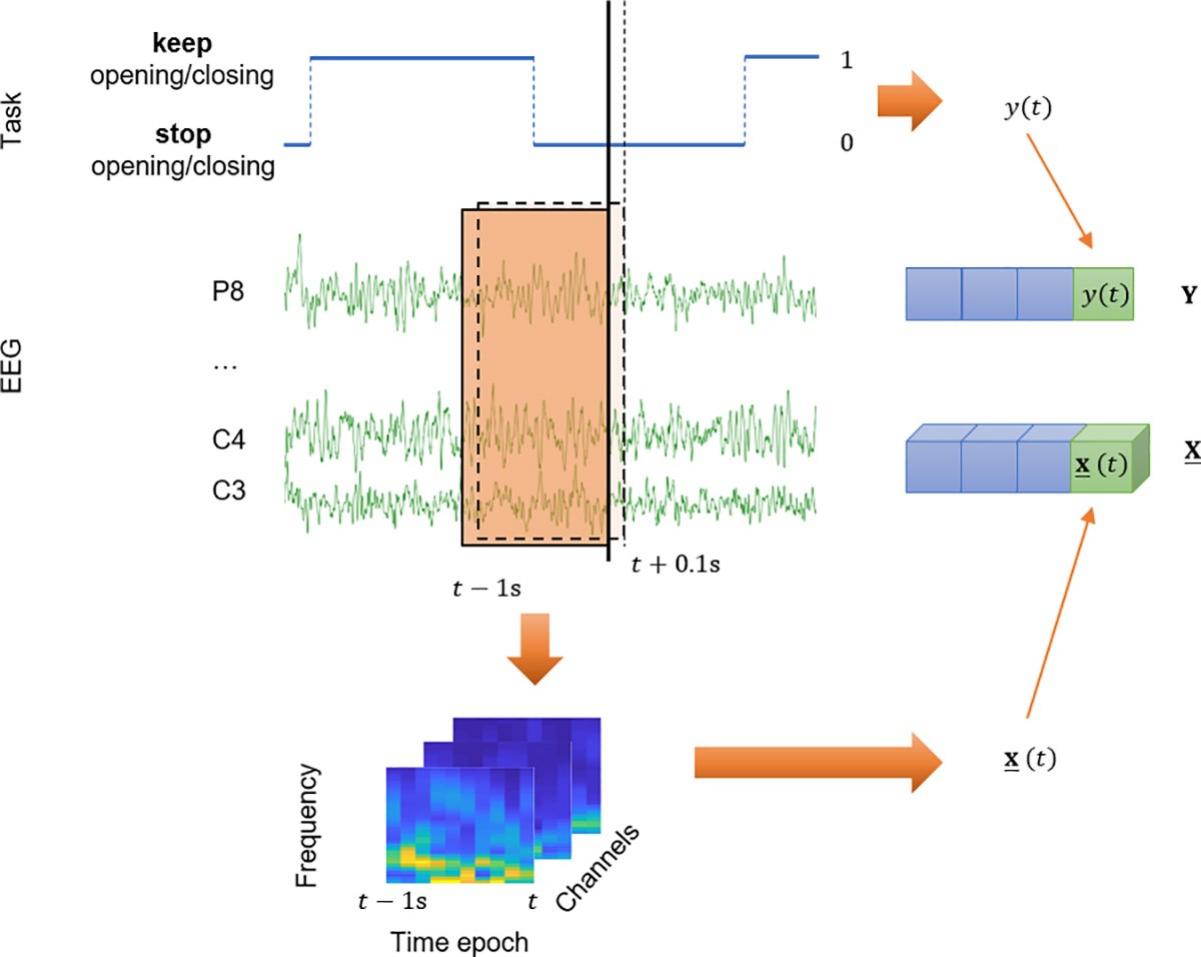
Data formation scheme. For each time *t*, to form the explanatory variable **x**(*t*), EEG signal from the electrodes was mapped by continuous wavelet transform with 35 frequencies (1, 2, …, 35 Hz). Then, the absolute values of the wavelet coefficients were decimated along the temporal modality to receive 10 points. The response variable *y*(*t*) was equal to 1 during the command “Keep opening and closing …” and was equal to 0 during the command “Stop opening and closing …”. The next epoch was taken with a time step of 100 ms.

The input data feature tensor **X** was formed from EEG epochs containing 1 second of the signal taken continuously with a time step of 100 ms [16, 17]. Each EEG epoch was mapped by the continuous wavelet transform (CWT) to the frequency-spatial-temporal feature space. The complex Morlet wavelet was chosen as a mother wavelet [17–19] and the analyzed frequency band ranged from 1 to 35 Hz with 1 Hz steps. Absolute values of the wavelet coefficients were decimated along the temporal domain by averaging 10 sliding windows each one of 100 ms long. The output variable *y* was equal to 1 or 0: 1 corresponded to the command “… keep opening and closing …”, and 0 corresponded to the command “… stop opening and closing …” (Fig 4).

### Signal decoding

**Fig 5:**
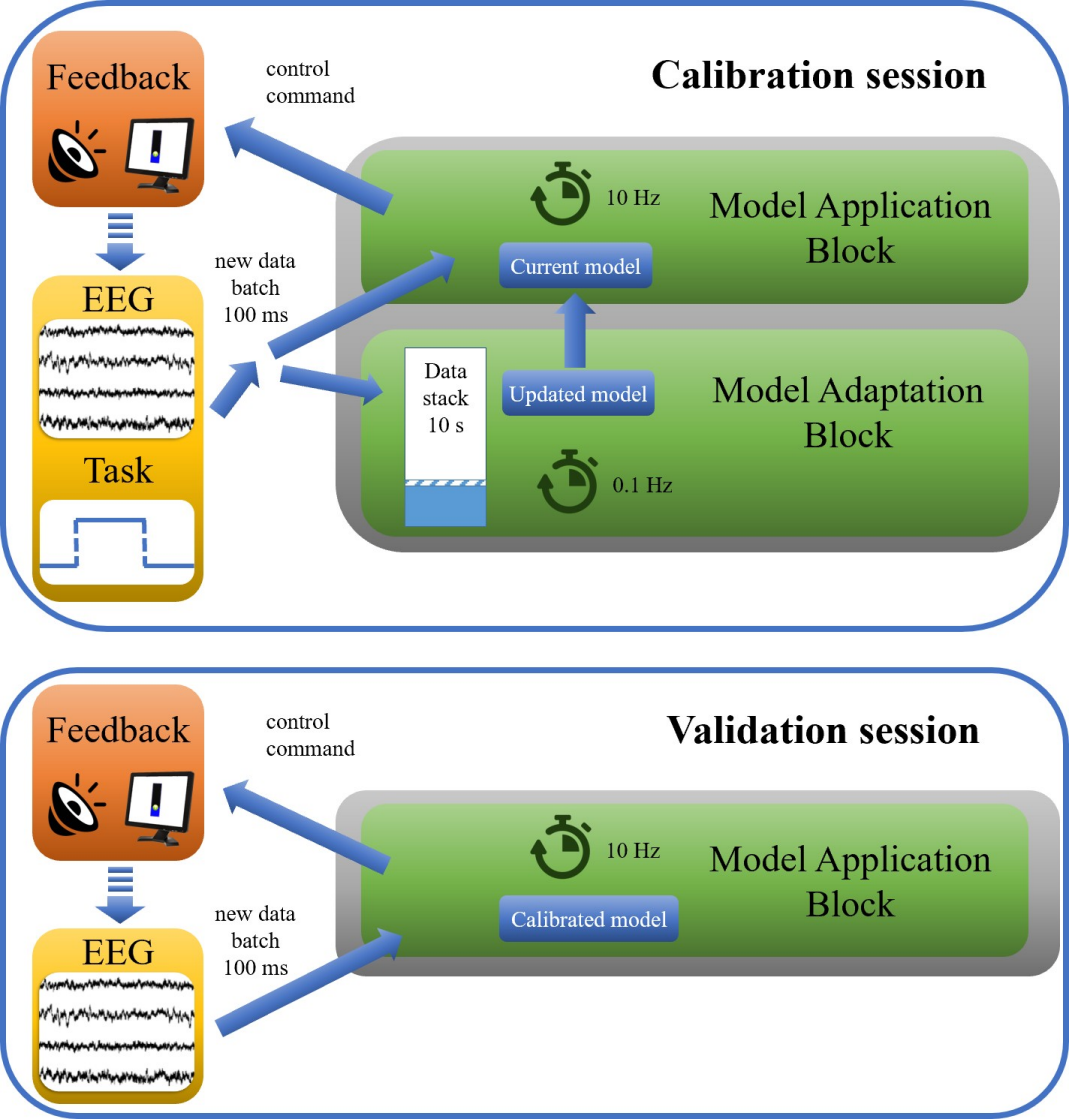
Signal decoding during calibration and validation sessions of the BCI experiment. The model was updated every 10 seconds during the calibration session and fixed during the validation session.

The REW-NPLS algorithm [16] was chosen for signal decoding as it demonstrated high efficiency in prior BCI applications [16, 17]. The decoding model created by the REW-NPLS algorithm was used to predict the response variable, i.e., the intention of opening and closing of the hand, every 100 ms. During the calibration session the model was adjusted every 10 seconds, while the model was fixed during the validation session (Fig 5).

### Quality evaluation

The quality of the subject’s control over the BCI system was estimated by correlation *r* between the predicted states and the real states. In addition, the True Positive Rate (TPR) and the True Negative Rate (TNR) were obtained. The TPR represents the proportion of the ‘… keep opening and closing …’ states that were correctly predicted. TNR represents correct prediction of the ‘… stop opening and closing …’ states. The BCI system is set to predict the state every 100 ms. To test significance, we created 1000 randomized models generated from the recorded data after randomly shuffling of the epoch labels. Significance of the correlation achieved by the predictive model was quantified by a one-tailed permutation test at the significance level of *α* = 0.001 [20]. This test compared the correlation coefficients provided by the analyzed model with coefficients generated by models based on random permutation of epoch labels.

## Results

We performed a total of 23 recordings using the self-paced BCI system, including 5 healthy volunteers, 14 conscious and 4 unconscious patients. Amongst patient recordings, 4 were placed for study purposes alone and 14 were already in place at the time of enrollment. None of the EEGs in patients or healthy volunteers showed seizures at the time of enrolment.

### Healthy volunteers

All healthy volunteers were able to control the BCI system with correlation coefficients significantly greater than the correlations provided by the random models (*p*<0.001) (Table 1). The ability to control the BCI system was seen in both the auditory and visual-auditory feedback paradigms.

**Table 1:** Characteristics of the healthy volunteers.

Healthy volunteer	Age/gender	BCI feedback	BCI activation		
			*r*	TPR	TNR
1	34/M	Auditory/Visual	**0.58**	0.92	0.66
2	37/M	Auditory/Visual	**0.61**	0.77	0.83
3	34/M	Auditory	**0.49**	0.83	0.64
4	25/M	Auditory	**0.11**	0.74	0.48
5	29/M	Auditory	**0.41**	0.77	0.86

* bold font represents significant superiority over the results of the random model control (*p*<0.001).

BCI, Brain-Computer Interface

*r*, correlation coefficient between the real and the ideal control

TPR, True Positive Rate

TNR, True Negative Rate

M, male.

### Patients

**Fig 6:**
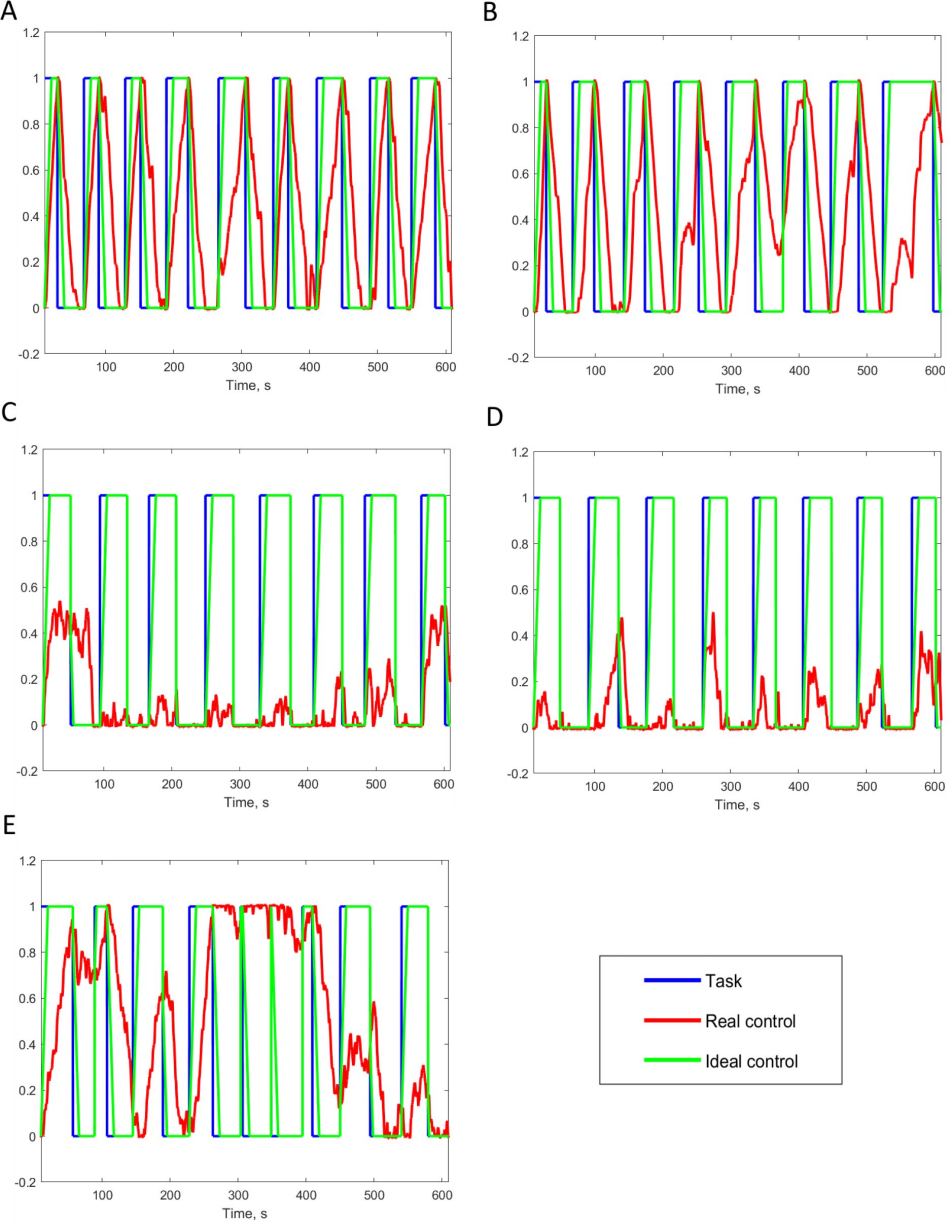
Testing results. (A) Patient 1, (B) Patient 2, (C) Patient 3, (D) Patient 4, (E) Patient 5. Both auditory and visual feedback were provided for the cases of (A) and (B); only auditory feedback was provided for the cases of (C), (D), and (E). For all the cases, value 1 of y-axis corresponds to the task “… keep opening and closing …”, and 0 corresponds to the task “… stop opening and closing …”.

The most common admission diagnoses were status epilepticus, ischemic stroke, subarachnoid hemorrhage, intracerebral hemorrhage, and subdural hematoma (Table 2). Of the behaviorally conscious patients, 7 had mild to moderate impairment of consciousness (1 anxiety, 6 lethargy) and 4 were comatose. Five of 14 conscious patients (36%) were able to control the provided BCI system using a motor command to move their hands, including one patient with quadriplegia. 2 of 3 conscious patients (67%) that were provided with auditory and visual feedback were able to control the BCI system. Three of 11 conscious patients (27%) that were tested using only auditory feedback were able to control the BCI system. For all five successful control cases, the correlation coefficients between the real and the ideal control were significantly greater than the correlation for the random control (*p*<0.001) (Table 2 and Fig 6). Among 9 conscious patients that were unable to control the BCI system, 6 were lethargic and 1 was anxious. None of the 4 unconscious patients were able to control the BCI system.

**Table 2:** Characteristics of the ICU patients.

Patient	Age/gender	Etiology	BCI feedback	BCI activation			CFS	Comments
				*r*	TPR	TNR		
Conscious								
1	65/M	SAH	Auditory/Visual	**0.64**	**0.9**	**0.8**	5	
2	28/F	C4-C8 fracture, spinal cord injury with quadriplegia	Auditory/Visual	**0.51**	**0.7**	**0.87**	5	
3	56/F	Neurotoxicity	Auditory	**0.28**	0.48	**0.6**	5	
4	60/M	Left temporal lobe ICH	Auditory	**0.29**	0.43	**0.57**	4	
5	75/M	Left thalamic/basal ganglia abscess	Auditory	**0.16**	**0.64**	0.5	5	
6	65/F	Nonconvulsive status epilepticus	Auditory	0.03	0.48	0.49	5	Anxious
7	88/M	Cardiac arrest, acute/early subacute pontine stroke	Auditory/Visual	-0.21	0.34	0.43	4	Lethargic
8	62/F	Central nervous system infection	Auditory	-0.06	0.44	0.47	4	Lethargic
9	61/M	Status epilepticus	Auditory	-0.1	0.35	0.34	4	Lethargic
10	75/M	SDH	Auditory	-0.14	0.5	0.29	5	
11	75/M	Left temporal ischemic stroke	Auditory	-0.23	0.42	0.38	4	
12	85/M	SDH	Auditory	-0.08	0.41	0.53	5	Lethargic
13	78/M	Right thalamic ICH	Auditory	-0.15	**0.74**	0.08	5	Lethargic
14	85/F	SAH	Auditory	-0.1	**0.69**	0.29	5	Lethargic
Unconscious								
15	84/M	SAH	Auditory	-0.06	**0.61**	0.31	1	
16	48/F	Nonconvulsive status epilepticus with SDH	Auditory	-0.1	0.45	0.47	2	
17	58/F	SAH, ICH	Auditory	-0.1	0.27	0.46	3	
18	61/M	Acute ischemic brainstem stroke	Auditory	-0.18	0.28	0.47	3	
Random model								
*N* = 1000, *r* = 0.00 ± 0.01, TPR = 0.50 ± 0.01, TNR = 0.50 ± 0.02								

* bold font represents significant superiority over the results of the random model control (*p*<0.001).

BCI, Brain-Computer Interface

*r*, correlation coefficient between the real and the ideal control

TPR, True Positive Rate

TNR, True Negative Rate

ICH, intracerebral hemorrhage

SAH, subarachnoid hemorrhage

SDH, subdural hemorrhage

M, male

F, female

CFS, Command Following Score: 1—coma, 2—eye opening, 3—tracking attending, 4—simple commands, 5—complex commands.

## Discussion

Current BCI systems developed for the ICU environment are cue-based. These systems are only active during pre-defined times that a cue is presented. In this study, we propose a prototype of a self-paced BCI system, which allows BCI activation when the patient wants to activate the system. Alerting health care providers to pain or discomfort will have much greater impact if patients are not restricted to prespecified time points. Here we report the initial development of our self-paced BCI system for ICU patients. The proposed self-paced prototype developed by our team utilizes a standard, clinical EEG system and a standard, commercially available computer to provide sensory feedback to the subject. This approach building on widely available technology will support more easy integration into clinical practice.

The experimental design consisted of a highly restricted number of experimental trials with only one or two sessions allowed for model calibration and a single model testing session. Conducting several trials on multiple days would have been preferable to optimize the model. We chose this approach due to unique challenges faced in patients with acute brain injury in the critical care setting. Fatigue that quickly develops in any patient undergoing repeated 5–10 minute lasting training and validation trials is magnified early after brain injury, in patients with pain, and the critical care context in general. We explored different numbers of training trials and found that we were able to get more reliable results using a minimum number of no more than two training sessions. We demonstrated that despite these challenging ICU conditions, several patients were able to gain control of the BCI system. The online calibration of the model, which allows immediate feedback to the patient constituted the principal factor for this success as this feedback enables the patient to rapidly develop a control strategy. Next we are planning to integrate this BCI supported feedback for detection of CMD, building on a recently published approach [21].

To quantify user control over the BCI system, we considered the TPR, which represents the proportion of ‘… keep opening and closing …’ states that are correctly identified, and the TNR, representing the proportion of ‘… stop opening and closing …’ states that are correctly identified. As these two parameters may dissociate, we also calculated the correlation coefficient between real and ideal control trajectories.

This system was tested both on healthy volunteers and patients, which were on a spectrum of impaired of consciousness. All heathy volunteers and 36% of conscious patients were able to reliably activate the BCI system using a motor intention even if they were unable to move their hand.

The study cohort presented here did not adequately reflect the full spectrum of patients with disorders of consciousness in the critical care setting and was underpowered to determine the potential benefits in unresponsive appearing patients with cognitive motor dissociation, a group for which BCI systems may have tremendous benefits. It demonstrated that, in the critical care context, it is possible to apply a BCI system built on widely available EEG technology in the clinical context. The study presented here illustrates the successful optimization and adjustment of the experimental protocol within the ICU context. As external noise may provide a challenge for BCI systems, future studies will focus on quantifying the impact of these on BCI success.

All five healthy volunteers demonstrated ability to reliably control the BCI system on the level that significantly outperformed the random model control. Both types of sensory feedback, auditory and auditory-visual, were tested on healthy volunteers. Although, the results and patient comments gave preference to auditory-visual feedback, the experiments have demonstrated that auditory feedback alone is also feasible. This is important, as development of a BCI system for patients in cognitive motor dissociation, who frequently have their eyes closed, is the long-term goal of this project. Some level of visual feedback, however, may be feasible even in patients that do not open their eyes such as light of different colors projected on the eyelids. This would be among the goals of future BCI studies.

Impairment of consciousness may involve impairment of comprehension as well as arousal [22], as impairment of arousal is a common in brain injured patients [23]. Our study also demonstrated that patients with disorders of arousal may have challenges in successfully utilizing the BCI system. The level of arousal which precludes successful BCI interaction is, however, uncertain. To determine the threshold of impaired arousal, all command following non-aphasic patients will be formally tested using the 60-Second Test [24]. In addition, it should be investigated if patients with cognitive motor dissociation that demonstrate command following on a functional MRI [2] or EEG assessment [1] are able to interact with the BCI system. The following studies will be devoted to solving these questions. The presented pilot study is a major step forward as it provides the foundation to carry out these experiments in the ICU environment.

### Limitations

We elected to only allow a small number of training sessions and validation sessions, but felt that it was important to show that the BCI system is able to work under these circumstances keeping concerns about patient fatigue in mind. Importantly, in behaviorally unresponsive patients it is particularly challenging to detect fatigue. Additionally, our cohort of healthy volunteers was not well matched in terms of gender or age distribution when compared to our patient cohort. This study was not conceptualized to comprehensively study if BCI activation in cognitive motor dissociation is possible but this question will be the focus of future studies utilizing the BCI system presented here.

In summary, our results show that conscious patients can achieve control over a self-paced BCI system in a noisy ICU environment full of distraction and noise. Based on these initial observations we cannot judge to what level patients will be able to utilize the BCI system to express and communicate their most basic needs to providers or loved ones. However, developing a self-paced BCI system which does not exist at this point in time for the ICU context would constitute a major step forward in providing these patients some degree of autonomy in a challenging situation. Future studies will need to optimize the self-paced BCI system, determine the ability to communicate needs to others, assess patient, surrogate, and health care provider satisfaction with the BCI system, determine the minimum threshold of fatigue allowable for successful BCI activation, and determine if patients that are behaviorally unresponsive such as those in cognitive motor dissociation are able to utilize this BCI system.
